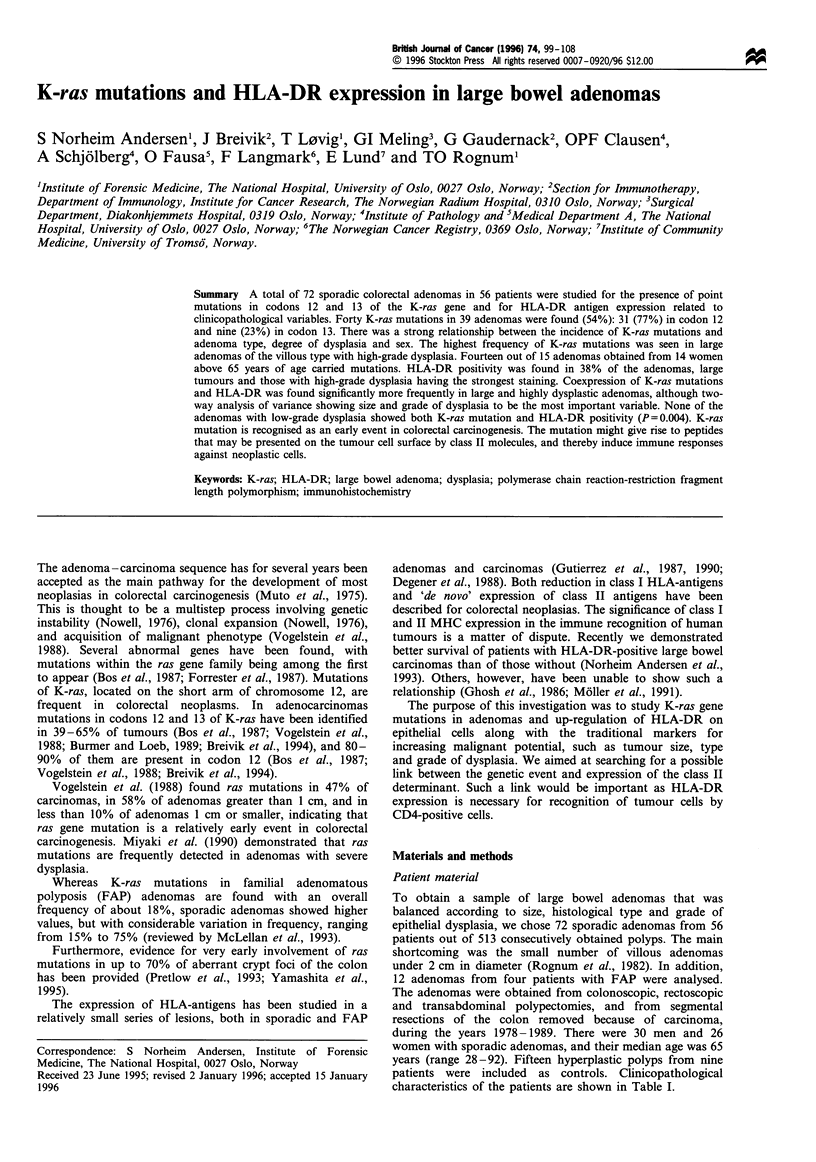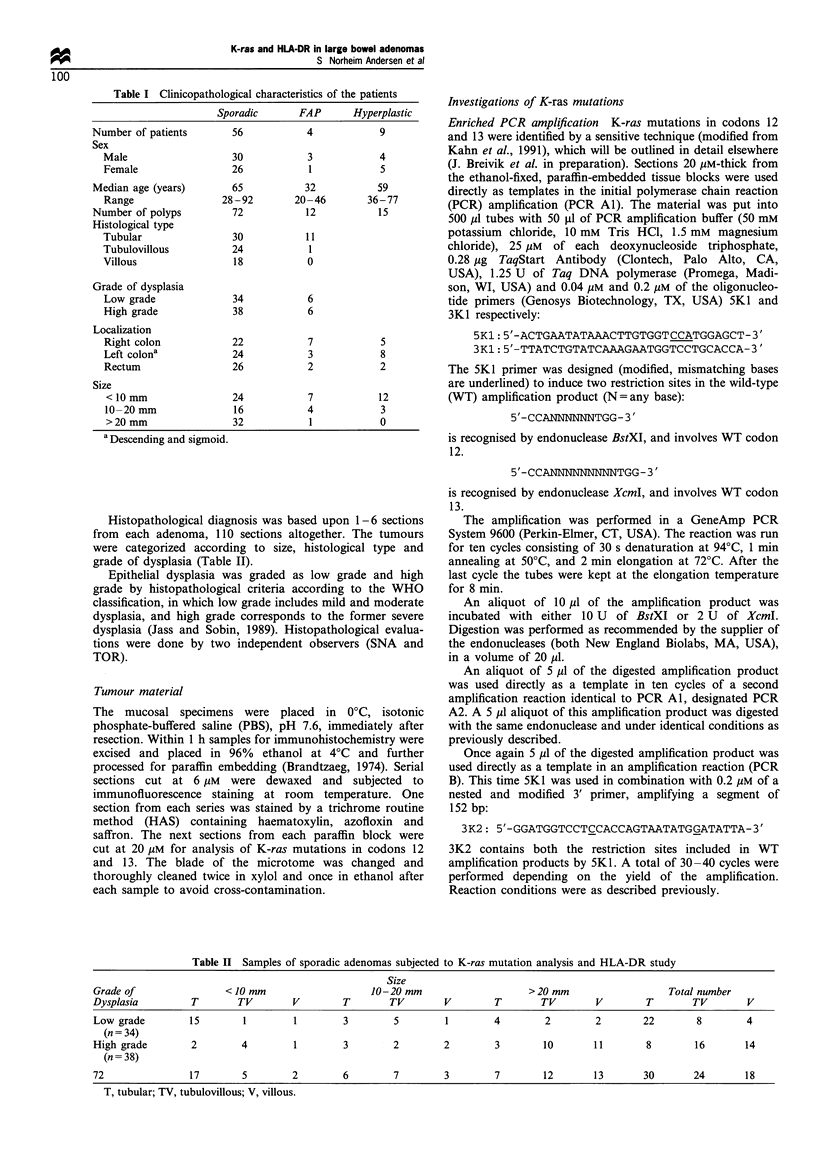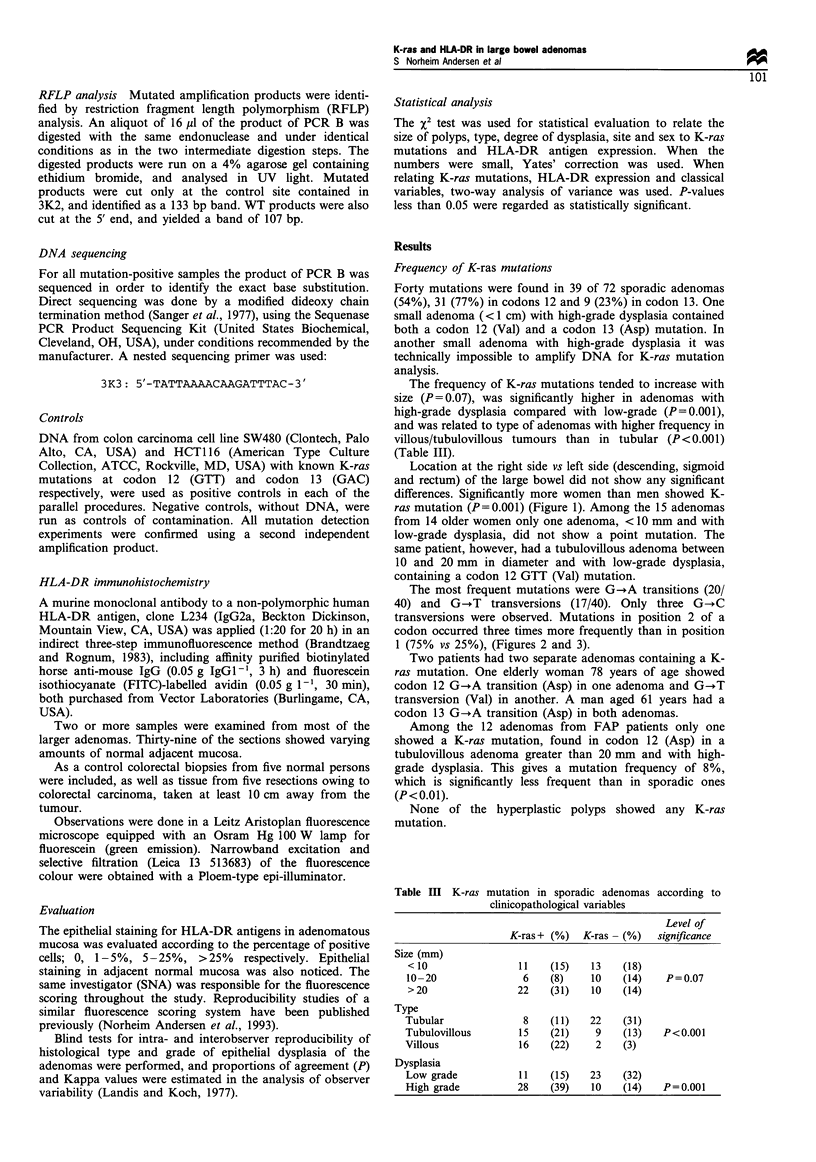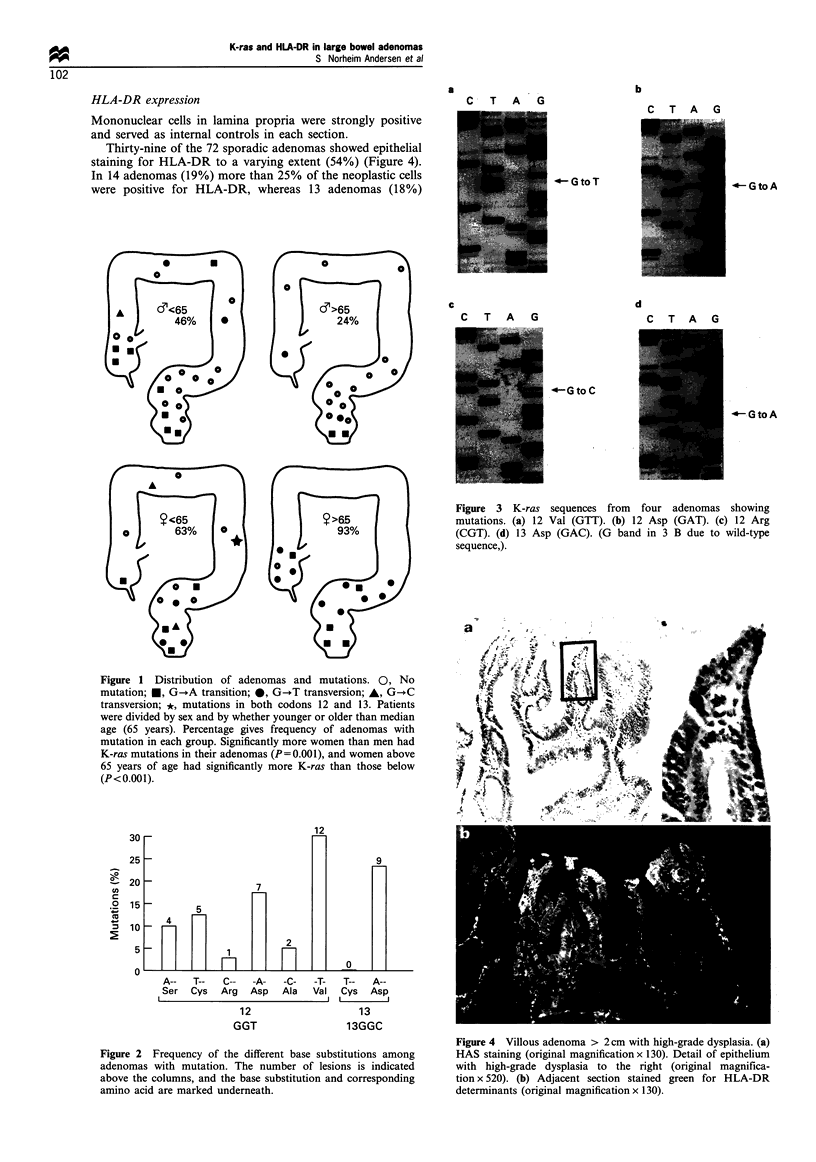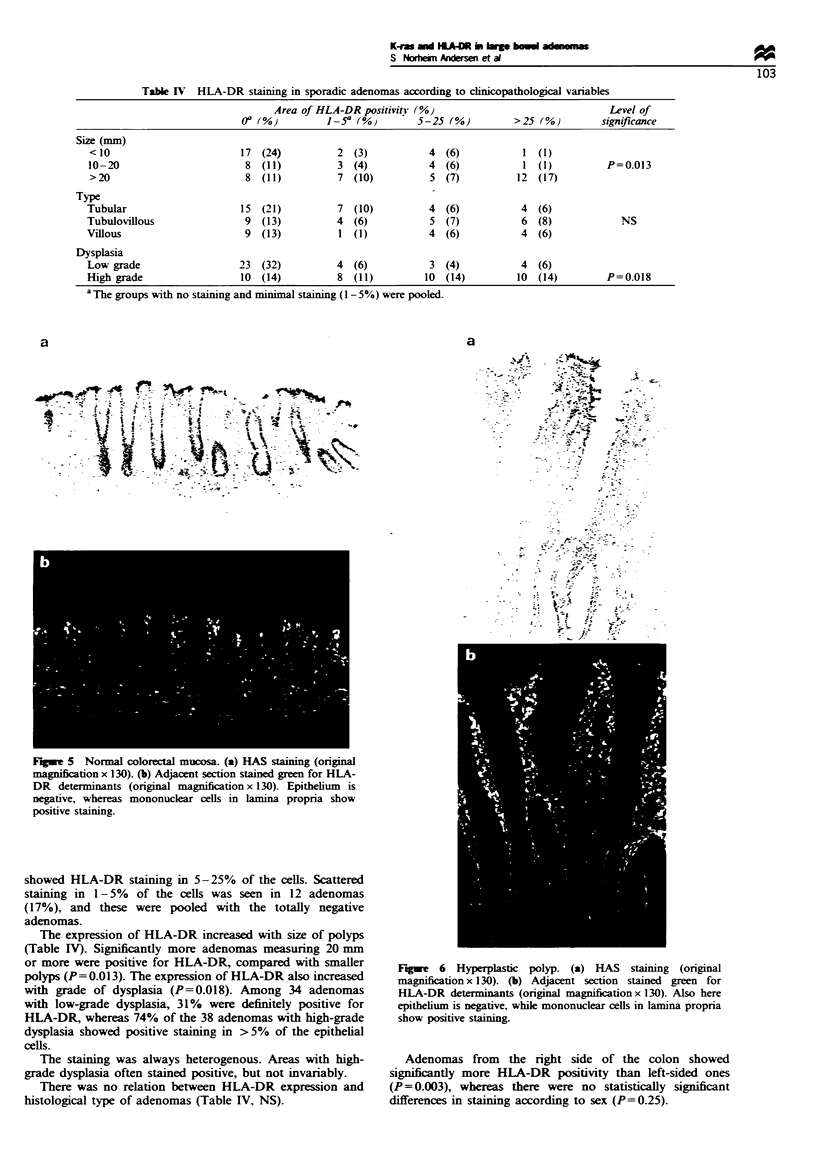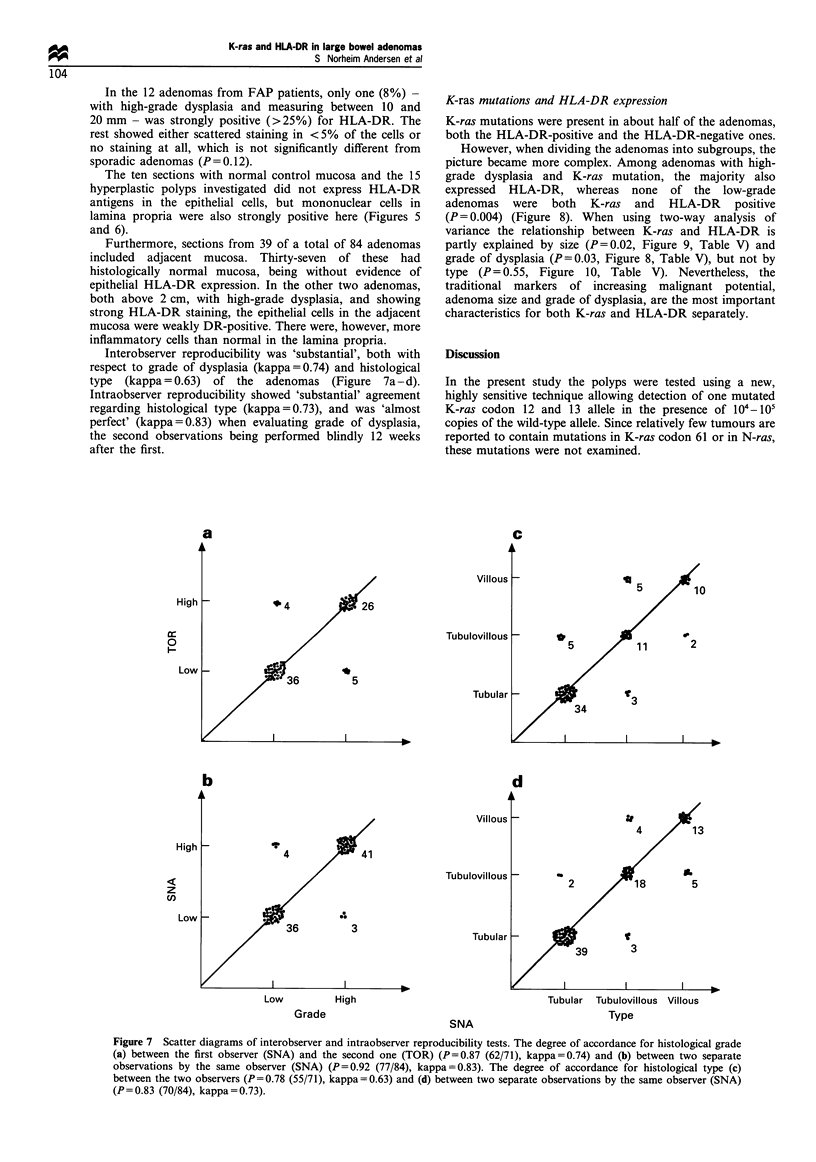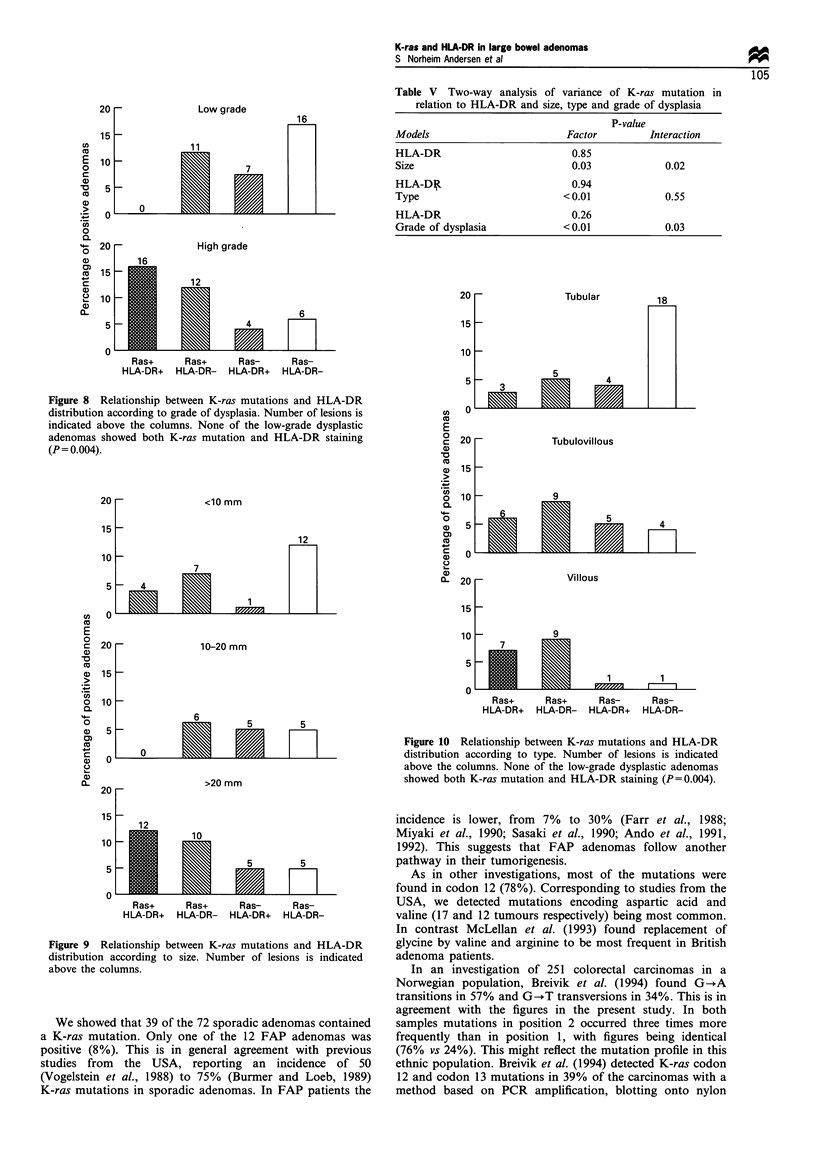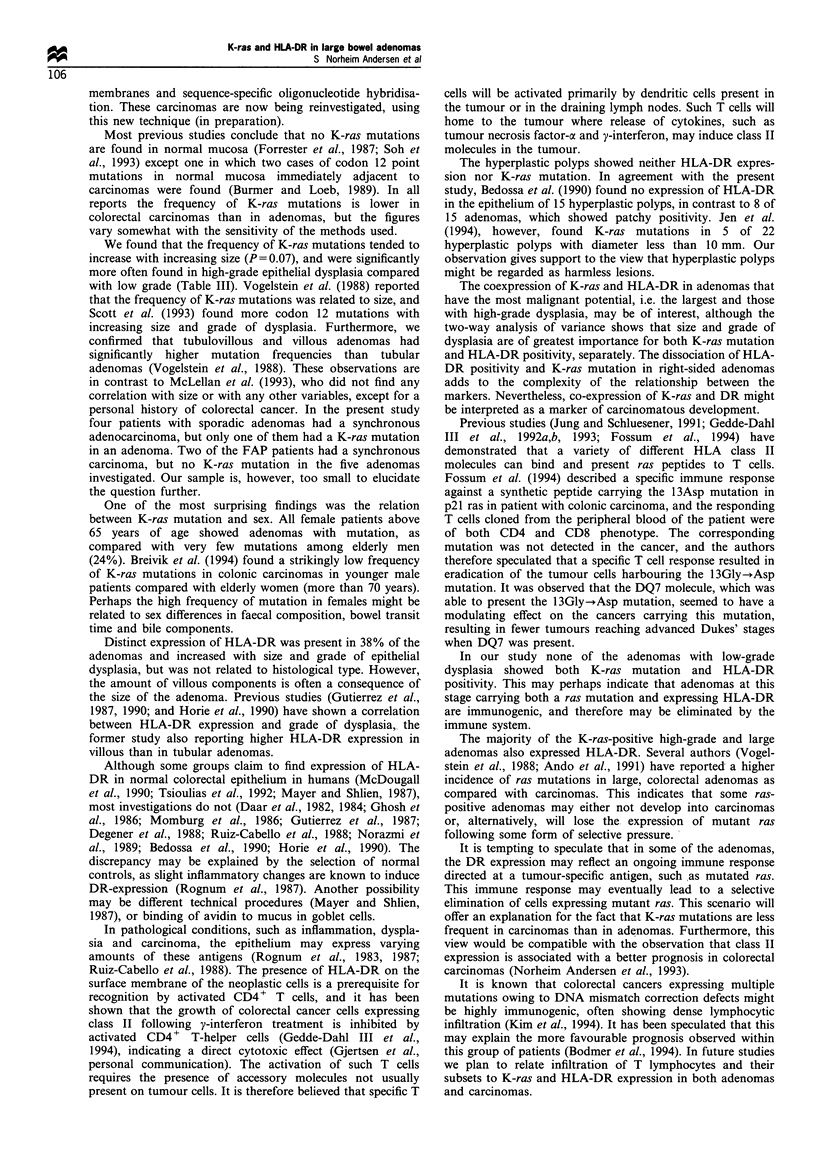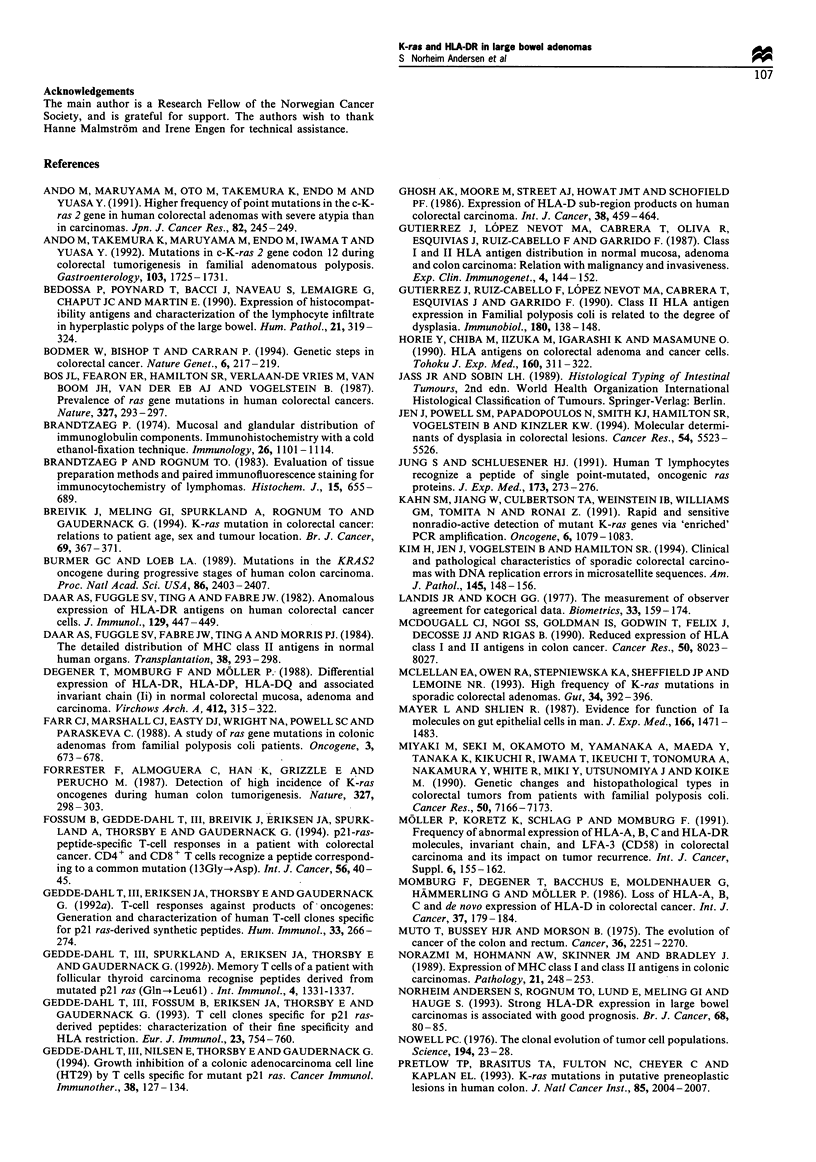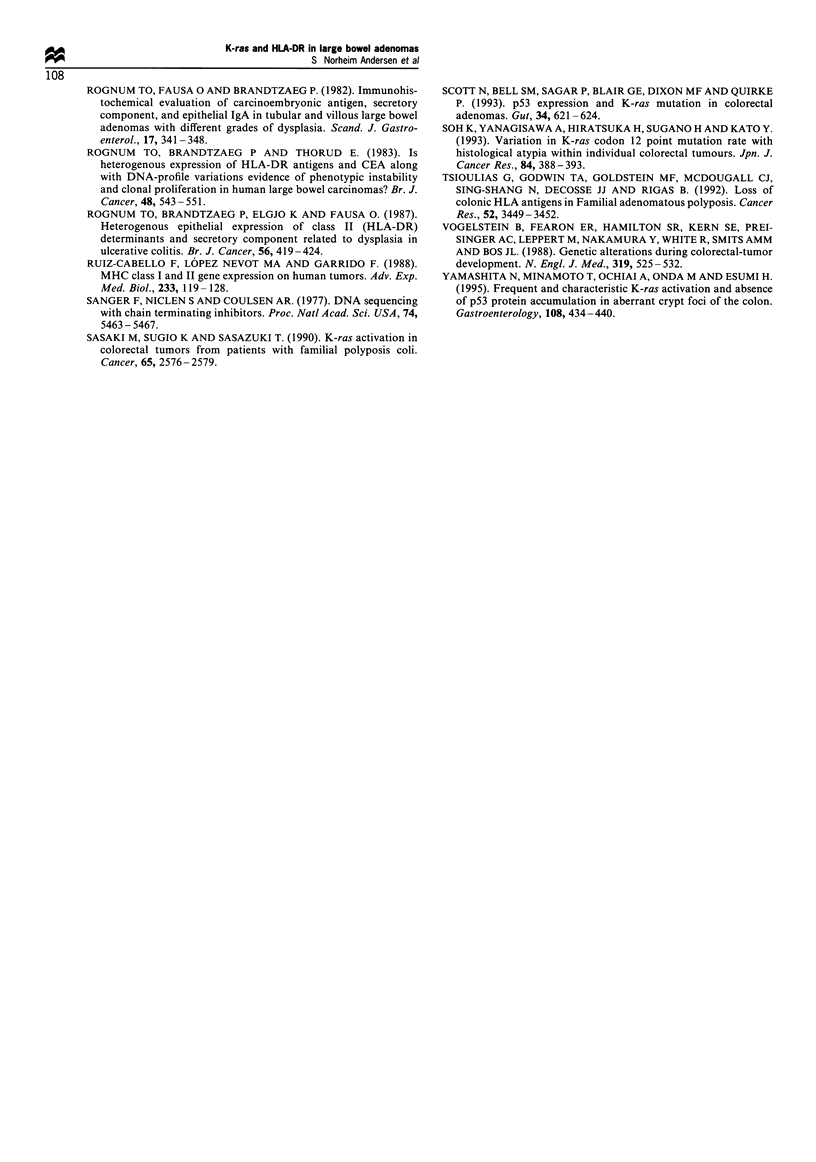# K-ras mutations and HLA-DR expression in large bowel adenomas.

**DOI:** 10.1038/bjc.1996.322

**Published:** 1996-07

**Authors:** S. Norheim Andersen, J. Breivik, T. Løvig, G. I. Meling, G. Gaudernack, O. P. Clausen, A. Schjölberg, O. Fausa, F. Langmark, E. Lund, T. O. Rognum

**Affiliations:** Institute of Forensic Medicine, National Hospital, University of Oslo, Norway.

## Abstract

**Images:**


British Journal of Cancer (1996) 74, 99-108
© 1996 Stockton Press All rights reserved 0007-0920/96 $12.00

### K-*ras* mutations and HLA-DR expression in large bowel adenomas

S Norheim Andersen[1], J Breivik[2], T Løvig[1], GI Meling[3], G Gaudernack[2], OPF Clausen[4], A Schjölberg[4], O Fausa[5], F Langmark[6], E Lund[7] and TO Rognum[1]

*[1]Institute of Forensic Medicine, The National Hospital, University of Oslo, 0027 Oslo, Norway; [2]Section for Immunotherapy, Department of Immunology, Institute for Cancer Research, The Norwegian Radium Hospital, 0310 Oslo, Norway; [3]Surgical Department, Diakonhjemmets Hospital, 0319 Oslo, Norway; [4]Institute of Pathology and [5]Medical Department A, The National Hospital, University of Oslo, 0027 Oslo, Norway; [6]The Norwegian Cancer Registry, 0369 Oslo, Norway; [7]Institute of Community Medicine, University of Tromsö, Norway.*

**Summary** A total of 72 sporadic colorectal adenomas in 56 patients were studied for the presence of point mutations in codons 12 and 13 of the K-*ras* gene and for HLA-DR antigen expression related to clinicopathological variables. Forty K-*ras* mutations in 39 adenomas were found (54%): 31 (77%) in codon 12 and nine (23%) in codon 13. There was a strong relationship between the incidence of K-*ras* mutations and adenoma type, degree of dysplasia and sex. The highest frequency of K-*ras* mutations was seen in large adenomas of the villous type with high-grade dysplasia. Fourteen out of 15 adenomas obtained from 14 women above 65 years of age carried mutations. HLA-DR positivity was found in 38% of the adenomas, large tumours and those with high-grade dysplasia having the strongest staining. Coexpression of K-*ras* mutations and HLA-DR was found significantly more frequently in large and highly dysplastic adenomas, although two-way analysis of variance showing size and grade of dysplasia to be the most important variable. None of the adenomas with low-grade dysplasia showed both K-*ras* mutation and HLA-DR positivity ($P=0.004$). K-*ras* mutation is recognised as an early event in colorectal carcinogenesis. The mutation might give rise to peptides that may be presented on the tumour cell surface by class II molecules, and thereby induce immune responses against neoplastic cells.

**Keywords:** K-*ras*; HLA-DR; large bowel adenoma; dysplasia; polymerase chain reaction-restriction fragment length polymorphism; immunohistochemistry

The adenoma–carcinoma sequence has for several years been accepted as the main pathway for the development of most neoplasias in colorectal carcinogenesis (Muto *et al.*, 1975). This is thought to be a multistep process involving genetic instability (Nowell, 1976), clonal expansion (Nowell, 1976), and acquisition of malignant phenotype (Vogelstein *et al.*, 1988). Several abnormal genes have been found, with mutations within the *ras* gene family being among the first to appear (Bos *et al.*, 1987; Forrester *et al.*, 1987). Mutations of K-*ras*, located on the short arm of chromosome 12, are frequent in colorectal neoplasms. In adenocarcinomas mutations in codons 12 and 13 of K-*ras* have been identified in 39–65% of tumours (Bos *et al.*, 1987; Vogelstein *et al.*, 1988; Burmer and Loeb, 1989; Breivik *et al.*, 1994), and 80–90% of them are present in codon 12 (Bos *et al.*, 1987; Vogelstein *et al.*, 1988; Breivik *et al.*, 1994).

Vogelstein *et al.* (1988) found *ras* mutations in 47% of carcinomas, in 58% of adenomas greater than 1 cm, and in less than 10% of adenomas 1 cm or smaller, indicating that *ras* gene mutation is a relatively early event in colorectal carcinogenesis. Miyaki *et al.* (1990) demonstrated that *ras* mutations are frequently detected in adenomas with severe dysplasia.

Whereas K-*ras* mutations in familial adenomatous polyposis (FAP) adenomas are found with an overall frequency of about 18%, sporadic adenomas showed higher values, but with considerable variation in frequency, ranging from 15% to 75% (reviewed by McLellan *et al.*, 1993).

Furthermore, evidence for very early involvement of *ras* mutations in up to 70% of aberrant crypt foci of the colon has been provided (Pretlow *et al.*, 1993; Yamashita *et al.*, 1995).

The expression of HLA-antigens has been studied in a relatively small series of lesions, both in sporadic and FAP adenomas and carcinomas (Gutierrez *et al.*, 1987, 1990; Degener *et al.*, 1988). Both reduction in class I HLA-antigens and '*de novo*' expression of class II antigens have been described for colorectal neoplasias. The significance of class I and II MHC expression in the immune recognition of human tumours is a matter of dispute. Recently we demonstrated better survival of patients with HLA-DR-positive large bowel carcinomas than of those without (Norheim Andersen *et al.*, 1993). Others, however, have been unable to show such a relationship (Ghosh *et al.*, 1986; Möller *et al.*, 1991).

The purpose of this investigation was to study K-*ras* gene mutations in adenomas and up-regulation of HLA-DR on epithelial cells along with the traditional markers for increasing malignant potential, such as tumour size, type and grade of dysplasia. We aimed at searching for a possible link between the genetic event and expression of the class II determinant. Such a link would be important as HLA-DR expression is necessary for recognition of tumour cells by CD4-positive cells.

### Materials and methods

### *Patient material*

To obtain a sample of large bowel adenomas that was balanced according to size, histological type and grade of epithelial dysplasia, we chose 72 sporadic adenomas from 56 patients out of 513 consecutively obtained polyps. The main shortcoming was the small number of villous adenomas under 2 cm in diameter (Rognum *et al.*, 1982). In addition, 12 adenomas from four patients with FAP were analysed. The adenomas were obtained from colonoscopic, rectoscopic and transabdominal polypectomies, and from segmental resections of the colon removed because of carcinoma, during the years 1978–1989. There were 30 men and 26 women with sporadic adenomas, and their median age was 65 years (range 28–92). Fifteen hyperplastic polyps from nine patients were included as controls. Clinicopathological characteristics of the patients are shown in Table I.

Correspondence: S Norheim Andersen, Institute of Forensic Medicine, The National Hospital, 0027 Oslo, Norway
Received 23 June 1995; revised 2 January 1996; accepted 15 January 1996

**Table I** Clinicopathological characteristics of the patients

| | *Sporadic* | *FAP* | *Hyperplastic* |
|---|---|---|---|
| Number of patients | 56 | 4 | 9 |
| Sex | | | |
| Male | 30 | 3 | 4 |
| Female | 26 | 1 | 5 |
| Median age (years) | 65 | 32 | 59 |
| Range | 28–92 | 20–46 | 36–77 |
| Number of polyps | 72 | 12 | 15 |
| Histological type | | | |
| Tubular | 30 | 11 | |
| Tubulovillous | 24 | 1 | |
| Villous | 18 | 0 | |
| Grade of dysplasia | | | |
| Low grade | 34 | 6 | |
| High grade | 38 | 6 | |
| Localization | | | |
| Right colon | 22 | 7 | 5 |
| Left colon[a] | 24 | 3 | 8 |
| Rectum | 26 | 2 | 2 |
| Size | | | |
| < 10 mm | 24 | 7 | 12 |
| 10–20 mm | 16 | 4 | 3 |
| > 20 mm | 32 | 1 | 0 |

[a] Descending and sigmoid.

Histopathological diagnosis was based upon 1–6 sections from each adenoma, 110 sections altogether. The tumours were categorized according to size, histological type and grade of dysplasia (Table II).

Epithelial dysplasia was graded as low grade and high grade by histopathological criteria according to the WHO classification, in which low grade includes mild and moderate dysplasia, and high grade corresponds to the former severe dysplasia (Jass and Sobin, 1989). Histopathological evaluations were done by two independent observers (SNA and TOR).

### *Tumour material*

The mucosal specimens were placed in 0°C, isotonic phosphate-buffered saline (PBS), pH 7.6, immediately after resection. Within 1 h samples for immunohistochemistry were excised and placed in 96% ethanol at 4°C and further processed for paraffin embedding (Brandtzaeg, 1974). Serial sections cut at 6 $\mu$M were dewaxed and subjected to immunofluorescence staining at room temperature. One section from each series was stained by a trichrome routine method (HAS) containing haematoxylin, azofloxin and saffron. The next sections from each paraffin block were cut at 20 $\mu$M for analysis of K-*ras* mutations in codons 12 and 13. The blade of the microtome was changed and thoroughly cleaned twice in xylol and once in ethanol after each sample to avoid cross-contamination.

### *Investigations of K-*ras *mutations*

*Enriched PCR amplification* K-*ras* mutations in codons 12 and 13 were identified by a sensitive technique (modified from Kahn *et al.*, 1991), which will be outlined in detail elsewhere (J. Breivik *et al.* in preparation). Sections 20 $\mu$M-thick from the ethanol-fixed, paraffin-embedded tissue blocks were used directly as templates in the initial polymerase chain reaction (PCR) amplification (PCR A1). The material was put into 500 $\mu$l tubes with 50 $\mu$l of PCR amplification buffer (50 mM potassium chloride, 10 mM Tris HCl, 1.5 mM magnesium chloride), 25 $\mu$M of each deoxynucleoside triphosphate, 0.28 $\mu$g *Taq*Start Antibody (Clontech, Palo Alto, CA, USA), 1.25 U of *Taq* DNA polymerase (Promega, Madison, WI, USA) and 0.04 $\mu$M and 0.2 $\mu$M of the oligonucleotide primers (Genosys Biotechnology, TX, USA) 5K1 and 3K1 respectively:

5K1 : 5′-ACTGAATATAAACTTGTGGTCCATGGAGCT-3′
3K1 : 5′-TTATCTGTATCAAAGAATGGTCCTGCACCA-3′

The 5K1 primer was designed (modified, mismatching bases are underlined) to induce two restriction sites in the wild-type (WT) amplification product (N = any base):

5′-CCANNNNNNTGG-3′

is recognised by endonuclease *Bst*XI, and involves WT codon 12.

5′-CCANNNNNNNNNTGG-3′

is recognised by endonuclease *Xcm*I, and involves WT codon 13.

The amplification was performed in a GeneAmp PCR System 9600 (Perkin-Elmer, CT, USA). The reaction was run for ten cycles consisting of 30 s denaturation at 94°C, 1 min annealing at 50°C, and 2 min elongation at 72°C. After the last cycle the tubes were kept at the elongation temperature for 8 min.

An aliquot of 10 $\mu$l of the amplification product was incubated with either 10 U of *Bst*XI or 2 U of *Xcm*I. Digestion was performed as recommended by the supplier of the endonucleases (both New England Biolabs, MA, USA), in a volume of 20 $\mu$l.

An aliquot of 5 $\mu$l of the digested amplification product was used directly as a template in ten cycles of a second amplification reaction identical to PCR A1, designated PCR A2. A 5 $\mu$l aliquot of this amplification product was digested with the same endonuclease and under identical conditions as previously described.

Once again 5 $\mu$l of the digested amplification product was used directly as a template in an amplification reaction (PCR B). This time 5K1 was used in combination with 0.2 $\mu$M of a nested and modified 3′ primer, amplifying a segment of 152 bp:

3K2 : 5′-GGATGGTCCTCCACCAGTAATATGGATATTA-3′

3K2 contains both the restriction sites included in WT amplification products by 5K1. A total of 30–40 cycles were performed depending on the yield of the amplification. Reaction conditions were as described previously.

**Table II** Samples of sporadic adenomas subjected to K-*ras* mutation analysis and HLA-DR study

| *Grade of* | Size: *< 10 mm* | | | *10–20 mm* | | | *> 20 mm* | | | *Total number* | | |
|---|---|---|---|---|---|---|---|---|---|---|---|---|
| *Dysplasia* | *T* | *TV* | *V* | *T* | *TV* | *V* | *T* | *TV* | *V* | *T* | *TV* | *V* |
| Low grade (*n* = 34) | 15 | 1 | 1 | 3 | 5 | 1 | 4 | 2 | 2 | 22 | 8 | 4 |
| High grade (*n* = 38) | 2 | 4 | 1 | 3 | 2 | 2 | 3 | 10 | 11 | 8 | 16 | 14 |
| 72 | 17 | 5 | 2 | 6 | 7 | 3 | 7 | 12 | 13 | 30 | 24 | 18 |

T, tubular; TV, tubulovillous; V, villous.

*RFLP analysis* Mutated amplification products were identified by restriction fragment length polymorphism (RFLP) analysis. An aliquot of 16 $\mu$l of the product of PCR B was digested with the same endonuclease and under identical conditions as in the two intermediate digestion steps. The digested products were run on a 4% agarose gel containing ethidium bromide, and analysed in UV light. Mutated products were cut only at the control site contained in 3K2, and identified as a 133 bp band. WT products were also cut at the 5′ end, and yielded a band of 107 bp.

*DNA sequencing*

For all mutation-positive samples the product of PCR B was sequenced in order to identify the exact base substitution. Direct sequencing was done by a modified dideoxy chain termination method (Sanger *et al*., 1977), using the Sequenase PCR Product Sequencing Kit (United States Biochemical, Cleveland, OH, USA), under conditions recommended by the manufacturer. A nested sequencing primer was used:

3K3: 5′-TATTAAAACAAGATTTAC-3′

*Controls*

DNA from colon carcinoma cell line SW480 (Clontech, Palo Alto, CA, USA) and HCT116 (American Type Culture Collection, ATCC, Rockville, MD, USA) with known K-*ras* mutations at codon 12 (GTT) and codon 13 (GAC) respectively, were used as positive controls in each of the parallel procedures. Negative controls, without DNA, were run as controls of contamination. All mutation detection experiments were confirmed using a second independent amplification product.

*HLA-DR immunohistochemistry*

A murine monoclonal antibody to a non-polymorphic human HLA-DR antigen, clone L234 (IgG2a, Beckton Dickinson, Mountain View, CA, USA) was applied (1:20 for 20 h) in an indirect three-step immunofluorescence method (Brandtzaeg and Rognum, 1983), including affinity purified biotinylated horse anti-mouse IgG (0.05 g $IgGl^{-1}$, 3 h) and fluorescein isothiocyanate (FITC)-labelled avidin (0.05 g $l^{-1}$, 30 min), both purchased from Vector Laboratories (Burlingame, CA, USA).

Two or more samples were examined from most of the larger adenomas. Thirty-nine of the sections showed varying amounts of normal adjacent mucosa.

As a control colorectal biopsies from five normal persons were included, as well as tissue from five resections owing to colorectal carcinoma, taken at least 10 cm away from the tumour.

Observations were done in a Leitz Aristoplan fluorescence microscope equipped with an Osram Hg 100 W lamp for fluorescein (green emission). Narrowband excitation and selective filtration (Leica I3 513683) of the fluorescence colour were obtained with a Ploem-type epi-illuminator.

*Evaluation*

The epithelial staining for HLA-DR antigens in adenomatous mucosa was evaluated according to the percentage of positive cells; 0, 1–5%, 5–25%, >25% respectively. Epithelial staining in adjacent normal mucosa was also noticed. The same investigator (SNA) was responsible for the fluorescence scoring throughout the study. Reproducibility studies of a similar fluorescence scoring system have been published previously (Norheim Andersen *et al*., 1993).

Blind tests for intra- and interobserver reproducibility of histological type and grade of epithelial dysplasia of the adenomas were performed, and proportions of agreement (*P*) and Kappa values were estimated in the analysis of observer variability (Landis and Koch, 1977).

*Statistical analysis*

The $\chi^2$ test was used for statistical evaluation to relate the size of polyps, type, degree of dysplasia, site and sex to K-*ras* mutations and HLA-DR antigen expression. When the numbers were small, Yates' correction was used. When relating K-*ras* mutations, HLA-DR expression and classical variables, two-way analysis of variance was used. *P*-values less than 0.05 were regarded as statistically significant.

### Results

*Frequency of K-*ras *mutations*

Forty mutations were found in 39 of 72 sporadic adenomas (54%), 31 (77%) in codons 12 and 9 (23%) in codon 13. One small adenoma (<1 cm) with high-grade dysplasia contained both a codon 12 (Val) and a codon 13 (Asp) mutation. In another small adenoma with high-grade dysplasia it was technically impossible to amplify DNA for K-*ras* mutation analysis.

The frequency of K-*ras* mutations tended to increase with size ($P=0.07$), was significantly higher in adenomas with high-grade dysplasia compared with low-grade ($P=0.001$), and was related to type of adenomas with higher frequency in villous/tubulovillous tumours than in tubular ($P<0.001$) (Table III).

Location at the right side *vs* left side (descending, sigmoid and rectum) of the large bowel did not show any significant differences. Significantly more women than men showed K-*ras* mutation ($P=0.001$) (Figure 1). Among the 15 adenomas from 14 older women only one adenoma, <10 mm and with low-grade dysplasia, did not show a point mutation. The same patient, however, had a tubulovillous adenoma between 10 and 20 mm in diameter and with low-grade dysplasia, containing a codon 12 GTT (Val) mutation.

The most frequent mutations were G→A transitions (20/40) and G→T transversions (17/40). Only three G→C transversions were observed. Mutations in position 2 of a codon occurred three times more frequently than in position 1 (75% *vs* 25%), (Figures 2 and 3).

Two patients had two separate adenomas containing a K-*ras* mutation. One elderly woman 78 years of age showed codon 12 G→A transition (Asp) in one adenoma and G→T transversion (Val) in another. A man aged 61 years had a codon 13 G→A transition (Asp) in both adenomas.

Among the 12 adenomas from FAP patients only one showed a K-*ras* mutation, found in codon 12 (Asp) in a tubulovillous adenoma greater than 20 mm and with high-grade dysplasia. This gives a mutation frequency of 8%, which is significantly less frequent than in sporadic ones ($P<0.01$).

None of the hyperplastic polyps showed any K-*ras* mutation.

**Table III** K-*ras* mutation in sporadic adenomas according to clinicopathological variables

| | *K*-ras+ | (%) | *K*-ras − | (%) | *Level of significance* |
|---|---|---|---|---|---|
| Size (mm) | | | | | |
| <10 | 11 | (15) | 13 | (18) | |
| 10–20 | 6 | (8) | 10 | (14) | $P=0.07$ |
| >20 | 22 | (31) | 10 | (14) | |
| Type | | | | | |
| Tubular | 8 | (11) | 22 | (31) | |
| Tubulovillous | 15 | (21) | 9 | (13) | $P<0.001$ |
| Villous | 16 | (22) | 2 | (3) | |
| Dysplasia | | | | | |
| Low grade | 11 | (15) | 23 | (32) | |
| High grade | 28 | (39) | 10 | (14) | $P=0.001$ |

### *HLA-DR expression*

Mononuclear cells in lamina propria were strongly positive and served as internal controls in each section.

Thirty-nine of the 72 sporadic adenomas showed epithelial staining for HLA-DR to a varying extent (54%) (Figure 4). In 14 adenomas (19%) more than 25% of the neoplastic cells were positive for HLA-DR, whereas 13 adenomas (18%)

**Figure 1** Distribution of adenomas and mutations. ○, No mutation; ■, G→A transition; ●, G→T transversion; ▲, G→C transversion; ★, mutations in both codons 12 and 13. Patients were divided by sex and by whether younger or older than median age (65 years). Percentage gives frequency of adenomas with mutation in each group. Significantly more women than men had K-*ras* mutations in their adenomas ($P = 0.001$), and women above 65 years of age had significantly more K-*ras* than those below ($P < 0.001$).

**Figure 2** Frequency of the different base substitutions among adenomas with mutation. The number of lesions is indicated above the columns, and the base substitution and corresponding amino acid are marked underneath.

**Figure 3** K-*ras* sequences from four adenomas showing mutations. (**a**) 12 Val (GTT). (**b**) 12 Asp (GAT). (**c**) 12 Arg (CGT). (**d**) 13 Asp (GAC). (G band in 3 B due to wild-type sequence,).

**Figure 4** Villous adenoma > 2 cm with high-grade dysplasia. (**a**) HAS staining (original magnification × 130). Detail of epithelium with high-grade dysplasia to the right (original magnification × 520). (**b**) Adjacent section stained green for HLA-DR determinants (original magnification × 130).

**Table IV** HLA-DR staining in sporadic adenomas according to clinicopathological variables

| | Area of HLA-DR positivity (%) $0^a$ (%) | $1-5^a$ (%) | 5–25 (%) | >25 (%) | Level of significance |
|---|---|---|---|---|---|
| Size (mm) | | | | | |
| <10 | 17 (24) | 2 (3) | 4 (6) | 1 (1) | |
| 10–20 | 8 (11) | 3 (4) | 4 (6) | 1 (1) | $P=0.013$ |
| >20 | 8 (11) | 7 (10) | 5 (7) | 12 (17) | |
| Type | | | | | |
| Tubular | 15 (21) | 7 (10) | 4 (6) | 4 (6) | |
| Tubulovillous | 9 (13) | 4 (6) | 5 (7) | 6 (8) | NS |
| Villous | 9 (13) | 1 (1) | 4 (6) | 4 (6) | |
| Dysplasia | | | | | |
| Low grade | 23 (32) | 4 (6) | 3 (4) | 4 (6) | |
| High grade | 10 (14) | 8 (11) | 10 (14) | 10 (14) | $P=0.018$ |

[a] The groups with no staining and minimal staining (1–5%) were pooled.

**Figure 5** Normal colorectal mucosa. (**a**) HAS staining (original magnification × 130). (**b**) Adjacent section stained green for HLA-DR determinants (original magnification × 130). Epithelium is negative, whereas mononuclear cells in lamina propria show positive staining.

**Figure 6** Hyperplastic polyp. (**a**) HAS staining (original magnification × 130). (**b**) Adjacent section stained green for HLA-DR determinants (original magnification × 130). Also here epithelium is negative, while mononuclear cells in lamina propria show positive staining.

showed HLA-DR staining in 5–25% of the cells. Scattered staining in 1–5% of the cells was seen in 12 adenomas (17%), and these were pooled with the totally negative adenomas.

The expression of HLA-DR increased with size of polyps (Table IV). Significantly more adenomas measuring 20 mm or more were positive for HLA-DR, compared with smaller polyps ($P=0.013$). The expression of HLA-DR also increased with grade of dysplasia ($P=0.018$). Among 34 adenomas with low-grade dysplasia, 31% were definitely positive for HLA-DR, whereas 74% of the 38 adenomas with high-grade dysplasia showed positive staining in >5% of the epithelial cells.

The staining was always heterogenous. Areas with high-grade dysplasia often stained positive, but not invariably.

There was no relation between HLA-DR expression and histological type of adenomas (Table IV, NS).

Adenomas from the right side of the colon showed significantly more HLA-DR positivity than left-sided ones ($P=0.003$), whereas there were no statistically significant differences in staining according to sex ($P=0.25$).

In the 12 adenomas from FAP patients, only one (8%) – with high-grade dysplasia and measuring between 10 and 20 mm – was strongly positive (>25%) for HLA-DR. The rest showed either scattered staining in <5% of the cells or no staining at all, which is not significantly different from sporadic adenomas ($P=0.12$).

The ten sections with normal control mucosa and the 15 hyperplastic polyps investigated did not express HLA-DR antigens in the epithelial cells, but mononuclear cells in lamina propria were also strongly positive here (Figures 5 and 6).

Furthermore, sections from 39 of a total of 84 adenomas included adjacent mucosa. Thirty-seven of these had histologically normal mucosa, being without evidence of epithelial HLA-DR expression. In the other two adenomas, both above 2 cm, with high-grade dysplasia, and showing strong HLA-DR staining, the epithelial cells in the adjacent mucosa were weakly DR-positive. There were, however, more inflammatory cells than normal in the lamina propria.

Interobserver reproducibility was 'substantial', both with respect to grade of dysplasia (kappa = 0.74) and histological type (kappa = 0.63) of the adenomas (Figure 7a–d). Intraobserver reproducibility showed 'substantial' agreement regarding histological type (kappa = 0.73), and was 'almost perfect' (kappa = 0.83) when evaluating grade of dysplasia, the second observations being performed blindly 12 weeks after the first.

*K*-ras *mutations and HLA-DR expression*

K-*ras* mutations were present in about half of the adenomas, both the HLA-DR-positive and the HLA-DR-negative ones.

However, when dividing the adenomas into subgroups, the picture became more complex. Among adenomas with high-grade dysplasia and K-*ras* mutation, the majority also expressed HLA-DR, whereas none of the low-grade adenomas were both K-*ras* and HLA-DR positive ($P=0.004$) (Figure 8). When using two-way analysis of variance the relationship between K-*ras* and HLA-DR is partly explained by size ($P=0.02$, Figure 9, Table V) and grade of dysplasia ($P=0.03$, Figure 8, Table V), but not by type ($P=0.55$, Figure 10, Table V). Nevertheless, the traditional markers of increasing malignant potential, adenoma size and grade of dysplasia, are the most important characteristics for both K-*ras* and HLA-DR separately.

### Discussion

In the present study the polyps were tested using a new, highly sensitive technique allowing detection of one mutated K-*ras* codon 12 and 13 allele in the presence of $10^4$–$10^5$ copies of the wild-type allele. Since relatively few tumours are reported to contain mutations in K-*ras* codon 61 or in N-*ras*, these mutations were not examined.

**Figure 7** Scatter diagrams of interobserver and intraobserver reproducibility tests. The degree of accordance for histological grade (**a**) between the first observer (SNA) and the second one (TOR) ($P=0.87$ (62/71), kappa = 0.74) and (**b**) between two separate observations by the same observer (SNA) ($P=0.92$ (77/84), kappa = 0.83). The degree of accordance for histological type (**c**) between the two observers ($P=0.78$ (55/71), kappa = 0.63) and (**d**) between two separate observations by the same observer (SNA) ($P=0.83$ (70/84), kappa = 0.73).

**Figure 8** Relationship between K-*ras* mutations and HLA-DR distribution according to grade of dysplasia. Number of lesions is indicated above the columns. None of the low-grade dysplastic adenomas showed both K-*ras* mutation and HLA-DR staining ($P=0.004$).

**Figure 9** Relationship between K-*ras* mutations and HLA-DR distribution according to size. Number of lesions is indicated above the columns.

We showed that 39 of the 72 sporadic adenomas contained a K-*ras* mutation. Only one of the 12 FAP adenomas was positive (8%). This is in general agreement with previous studies from the USA, reporting an incidence of 50 (Vogelstein *et al.*, 1988) to 75% (Burmer and Loeb, 1989) K-*ras* mutations in sporadic adenomas. In FAP patients the

**Table V** Two-way analysis of variance of K-*ras* mutation in relation to HLA-DR and size, type and grade of dysplasia

| | P-*value* | |
|---|---|---|
| *Models* | *Factor* | *Interaction* |
| HLA-DR | 0.85 | |
| Size | 0.03 | 0.02 |
| HLA-DR | 0.94 | |
| Type | <0.01 | 0.55 |
| HLA-DR | 0.26 | |
| Grade of dysplasia | <0.01 | 0.03 |

**Figure 10** Relationship between K-*ras* mutations and HLA-DR distribution according to type. Number of lesions is indicated above the columns. None of the low-grade dysplastic adenomas showed both K-*ras* mutation and HLA-DR staining ($P=0.004$).

incidence is lower, from 7% to 30% (Farr *et al.*, 1988; Miyaki *et al.*, 1990; Sasaki *et al.*, 1990; Ando *et al.*, 1991, 1992). This suggests that FAP adenomas follow another pathway in their tumorigenesis.

As in other investigations, most of the mutations were found in codon 12 (78%). Corresponding to studies from the USA, we detected mutations encoding aspartic acid and valine (17 and 12 tumours respectively) being most common. In contrast McLellan *et al.* (1993) found replacement of glycine by valine and arginine to be most frequent in British adenoma patients.

In an investigation of 251 colorectal carcinomas in a Norwegian population, Breivik *et al.* (1994) found G→A transitions in 57% and G→T transversions in 34%. This is in agreement with the figures in the present study. In both samples mutations in position 2 occurred three times more frequently than in position 1, with figures being identical (76% *vs* 24%). This might reflect the mutation profile in this ethnic population. Breivik *et al.* (1994) detected K-*ras* codon 12 and codon 13 mutations in 39% of the carcinomas with a method based on PCR amplification, blotting onto nylon membranes and sequence-specific oligonucleotide hybridisation. These carcinomas are now being reinvestigated, using this new technique (in preparation).

Most previous studies conclude that no K-*ras* mutations are found in normal mucosa (Forrester *et al.*, 1987; Soh *et al.*, 1993) except one in which two cases of codon 12 point mutations in normal mucosa immediately adjacent to carcinomas were found (Burmer and Loeb, 1989). In all reports the frequency of K-*ras* mutations is lower in colorectal carcinomas than in adenomas, but the figures vary somewhat with the sensitivity of the methods used.

We found that the frequency of K-*ras* mutations tended to increase with increasing size ($P = 0.07$), and were significantly more often found in high-grade epithelial dysplasia compared with low grade (Table III). Vogelstein *et al.* (1988) reported that the frequency of K-*ras* mutations was related to size, and Scott *et al.* (1993) found more codon 12 mutations with increasing size and grade of dysplasia. Furthermore, we confirmed that tubulovillous and villous adenomas had significantly higher mutation frequencies than tubular adenomas (Vogelstein *et al.*, 1988). These observations are in contrast to McLellan *et al.* (1993), who did not find any correlation with size or with any other variables, except for a personal history of colorectal cancer. In the present study four patients with sporadic adenomas had a synchronous adenocarcinoma, but only one of them had a K-*ras* mutation in an adenoma. Two of the FAP patients had a synchronous carcinoma, but no K-*ras* mutation in the five adenomas investigated. Our sample is, however, too small to elucidate the question further.

One of the most surprising findings was the relation between K-*ras* mutation and sex. All female patients above 65 years of age showed adenomas with mutation, as compared with very few mutations among elderly men (24%). Breivik *et al.* (1994) found a strikingly low frequency of K-*ras* mutations in colonic carcinomas in younger male patients compared with elderly women (more than 70 years). Perhaps the high frequency of mutation in females might be related to sex differences in faecal composition, bowel transit time and bile components.

Distinct expression of HLA-DR was present in 38% of the adenomas and increased with size and grade of epithelial dysplasia, but was not related to histological type. However, the amount of villous components is often a consequence of the size of the adenoma. Previous studies (Gutierrez *et al.*, 1987, 1990; and Horie *et al.*, 1990) have shown a correlation between HLA-DR expression and grade of dysplasia, the former study also reporting higher HLA-DR expression in villous than in tubular adenomas.

Although some groups claim to find expression of HLA-DR in normal colorectal epithelium in humans (McDougall *et al.*, 1990; Tsioulias *et al.*, 1992; Mayer and Shlien, 1987), most investigations do not (Daar *et al.*, 1982, 1984; Ghosh *et al.*, 1986; Momburg *et al.*, 1986; Gutierrez *et al.*, 1987; Degener *et al.*, 1988; Ruiz-Cabello *et al.*, 1988; Norazmi *et al.*, 1989; Bedossa *et al.*, 1990; Horie *et al.*, 1990). The discrepancy may be explained by the selection of normal controls, as slight inflammatory changes are known to induce DR-expression (Rognum *et al.*, 1987). Another possibility may be different technical procedures (Mayer and Shlien, 1987), or binding of avidin to mucus in goblet cells.

In pathological conditions, such as inflammation, dysplasia and carcinoma, the epithelium may express varying amounts of these antigens (Rognum *et al.*, 1983, 1987; Ruiz-Cabello *et al.*, 1988). The presence of HLA-DR on the surface membrane of the neoplastic cells is a prerequisite for recognition by activated $CD4^+$ T cells, and it has been shown that the growth of colorectal cancer cells expressing class II following $\gamma$-interferon treatment is inhibited by activated $CD4^+$ T-helper cells (Gedde-Dahl III *et al.*, 1994), indicating a direct cytotoxic effect (Gjertsen *et al.*, personal communication). The activation of such T cells requires the presence of accessory molecules not usually present on tumour cells. It is therefore believed that specific T cells will be activated primarily by dendritic cells present in the tumour or in the draining lymph nodes. Such T cells will home to the tumour where release of cytokines, such as tumour necrosis factor-$\alpha$ and $\gamma$-interferon, may induce class II molecules in the tumour.

The hyperplastic polyps showed neither HLA-DR expression nor K-*ras* mutation. In agreement with the present study, Bedossa *et al.* (1990) found no expression of HLA-DR in the epithelium of 15 hyperplastic polyps, in contrast to 8 of 15 adenomas, which showed patchy positivity. Jen *et al.* (1994), however, found K-*ras* mutations in 5 of 22 hyperplastic polyps with diameter less than 10 mm. Our observation gives support to the view that hyperplastic polyps might be regarded as harmless lesions.

The coexpression of K-*ras* and HLA-DR in adenomas that have the most malignant potential, i.e. the largest and those with high-grade dysplasia, may be of interest, although the two-way analysis of variance shows that size and grade of dysplasia are of greatest importance for both K-*ras* mutation and HLA-DR positivity, separately. The dissociation of HLA-DR positivity and K-*ras* mutation in right-sided adenomas adds to the complexity of the relationship between the markers. Nevertheless, co-expression of K-*ras* and DR might be interpreted as a marker of carcinomatous development.

Previous studies (Jung and Schluesener, 1991; Gedde-Dahl III *et al.*, 1992*a*,*b*, 1993; Fossum *et al.*, 1994) have demonstrated that a variety of different HLA class II molecules can bind and present *ras* peptides to T cells. Fossum *et al.* (1994) described a specific immune response against a synthetic peptide carrying the 13Asp mutation in p21 ras in patient with colonic carcinoma, and the responding T cells cloned from the peripheral blood of the patient were of both CD4 and CD8 phenotype. The corresponding mutation was not detected in the cancer, and the authors therefore speculated that a specific T cell response resulted in eradication of the tumour cells harbouring the 13Gly→Asp mutation. It was observed that the DQ7 molecule, which was able to present the 13Gly→Asp mutation, seemed to have a modulating effect on the cancers carrying this mutation, resulting in fewer tumours reaching advanced Dukes' stages when DQ7 was present.

In our study none of the adenomas with low-grade dysplasia showed both K-*ras* mutation and HLA-DR positivity. This may perhaps indicate that adenomas at this stage carrying both a *ras* mutation and expressing HLA-DR are immunogenic, and therefore may be eliminated by the immune system.

The majority of the K-*ras*-positive high-grade and large adenomas also expressed HLA-DR. Several authors (Vogelstein *et al.*, 1988; Ando *et al.*, 1991) have reported a higher incidence of *ras* mutations in large, colorectal adenomas as compared with carcinomas. This indicates that some *ras*-positive adenomas may either not develop into carcinomas or, alternatively, will lose the expression of mutant *ras* following some form of selective pressure.

It is tempting to speculate that in some of the adenomas, the DR expression may reflect an ongoing immune response directed at a tumour-specific antigen, such as mutated *ras*. This immune response may eventually lead to a selective elimination of cells expressing mutant *ras*. This scenario will offer an explanation for the fact that K-*ras* mutations are less frequent in carcinomas than in adenomas. Furthermore, this view would be compatible with the observation that class II expression is associated with a better prognosis in colorectal carcinomas (Norheim Andersen *et al.*, 1993).

It is known that colorectal cancers expressing multiple mutations owing to DNA mismatch correction defects might be highly immunogenic, often showing dense lymphocytic infiltration (Kim *et al.*, 1994). It has been speculated that this may explain the more favourable prognosis observed within this group of patients (Bodmer *et al.*, 1994). In future studies we plan to relate infiltration of T lymphocytes and their subsets to K-*ras* and HLA-DR expression in both adenomas and carcinomas.

**Acknowledgements**

The main author is a Research Fellow of the Norwegian Cancer Society, and is grateful for support. The authors wish to thank Hanne Malmström and Irene Engen for technical assistance.

### References

ANDO M, MARUYAMA M, OTO M, TAKEMURA K, ENDO M AND YUASA Y. (1991). Higher frequency of point mutations in the c-K-*ras 2* gene in human colorectal adenomas with severe atypia than in carcinomas. *Jpn. J. Cancer Res.*, **82,** 245–249.

ANDO M, TAKEMURA K, MARUYAMA M, ENDO M, IWAMA T AND YUASA Y. (1992). Mutations in c-K-*ras 2* gene codon 12 during colorectal tumorigenesis in familial adenomatous polyposis. *Gastroenterology*, **103,** 1725–1731.

BEDOSSA P, POYNARD T, BACCI J, NAVEAU S, LEMAIGRE G, CHAPUT JC AND MARTIN E. (1990). Expression of histocompatibility antigens and characterization of the lymphocyte infiltrate in hyperplastic polyps of the large bowel. *Hum. Pathol.*, **21,** 319–324.

BODMER W, BISHOP T AND CARRAN P. (1994). Genetic steps in colorectal cancer. *Nature Genet.*, **6,** 217–219.

BOS JL, FEARON ER, HAMILTON SR, VERLAAN-DE VRIES M, VAN BOOM JH, VAN DER EB AJ AND VOGELSTEIN B. (1987). Prevalence of *ras* gene mutations in human colorectal cancers. *Nature*, **327,** 293–297.

BRANDTZAEG P. (1974). Mucosal and glandular distribution of immunoglobulin components. Immunohistochemistry with a cold ethanol-fixation technique. *Immunology*, **26,** 1101–1114.

BRANDTZAEG P AND ROGNUM TO. (1983). Evaluation of tissue preparation methods and paired immunofluorescence staining for immunocytochemistry of lymphomas. *Histochem. J.*, **15,** 655–689.

BREIVIK J, MELING GI, SPURKLAND A, ROGNUM TO AND GAUDERNACK G. (1994). K-*ras* mutation in colorectal cancer: relations to patient age, sex and tumour location. *Br. J. Cancer*, **69,** 367–371.

BURMER GC AND LOEB LA. (1989). Mutations in the *KRAS2* oncogene during progressive stages of human colon carcinoma. *Proc. Natl Acad. Sci. USA*, **86,** 2403–2407.

DAAR AS, FUGGLE SV, TING A AND FABRE JW. (1982). Anomalous expression of HLA-DR antigens on human colorectal cancer cells. *J. Immunol.*, **129,** 447–449.

DAAR AS, FUGGLE SV, FABRE JW, TING A AND MORRIS PJ. (1984). The detailed distribution of MHC class II antigens in normal human organs. *Transplantation*, **38,** 293–298.

DEGENER T, MOMBURG F AND MÖLLER P. (1988). Differential expression of HLA-DR, HLA-DP, HLA-DQ and associated invariant chain (Ii) in normal colorectal mucosa, adenoma and carcinoma. *Virchows Arch. A*, **412,** 315–322.

FARR CJ, MARSHALL CJ, EASTY DJ, WRIGHT NA, POWELL SC AND PARASKEVA C. (1988). A study of *ras* gene mutations in colonic adenomas from familial polyposis coli patients. *Oncogene*, **3,** 673–678.

FORRESTER F, ALMOGUERA C, HAN K, GRIZZLE E AND PERUCHO M. (1987). Detection of high incidence of K-*ras* oncogenes during human colon tumorigenesis. *Nature*, **327,** 298–303.

FOSSUM B, GEDDE-DAHL T, III, BREIVIK J, ERIKSEN JA, SPURKLAND A, THORSBY E AND GAUDERNACK G. (1994). p21-*ras*-peptide-specific T-cell responses in a patient with colorectal cancer. $CD4^+$ and $CD8^+$ T cells recognize a peptide corresponding to a common mutation (13Gly→Asp). *Int. J. Cancer*, **56,** 40–45.

GEDDE-DAHL T, III, ERIKSEN JA, THORSBY E AND GAUDERNACK G. (1992*a*). T-cell responses against products of oncogenes: Generation and characterization of human T-cell clones specific for p21 *ras*-derived synthetic peptides. *Hum. Immunol.*, **33,** 266–274.

GEDDE-DAHL T, III, SPURKLAND A, ERIKSEN JA, THORSBY E AND GAUDERNACK G. (1992*b*). Memory T cells of a patient with follicular thyroid carcinoma recognise peptides derived from mutated p21 *ras* (Gln→Leu61) . *Int. Immunol.*, **4,** 1331-1337.

GEDDE-DAHL T, III, FOSSUM B, ERIKSEN JA, THORSBY E AND GAUDERNACK G. (1993). T cell clones specific for p21 *ras*-derived peptides: characterization of their fine specificity and HLA restriction. *Eur. J. Immunol.*, **23,** 754–760.

GEDDE-DAHL T, III, NILSEN E, THORSBY E AND GAUDERNACK G. (1994). Growth inhibition of a colonic adenocarcinoma cell line (HT29) by T cells specific for mutant p21 *ras*. *Cancer Immunol. Immunother.*, **38,** 127–134.

GHOSH AK, MOORE M, STREET AJ, HOWAT JMT AND SCHOFIELD PF. (1986). Expression of HLA-D sub-region products on human colorectal carcinoma. *Int. J. Cancer*, **38,** 459–464.

GUTIERREZ J, LÓPEZ NEVOT MA, CABRERA T, OLIVA R, ESQUIVIAS J, RUIZ-CABELLO F AND GARRIDO F. (1987). Class I and II HLA antigen distribution in normal mucosa, adenoma and colon carcinoma: Relation with malignancy and invasiveness. *Exp. Clin. Immunogenet.*, **4,** 144–152.

GUTIERREZ J, RUIZ-CABELLO F, LÓPEZ NEVOT MA, CABRERA T, ESQUIVIAS J AND GARRIDO F. (1990). Class II HLA antigen expression in Familial polyposis coli is related to the degree of dysplasia. *Immunobiol.*, **180,** 138–148.

HORIE Y, CHIBA M, IIZUKA M, IGARASHI K AND MASAMUNE O. (1990). HLA antigens on colorectal adenoma and cancer cells. *Tohoku J. Exp. Med.*, **160,** 311–322.

JASS JR AND SOBIN LH. (1989). *Histological Typing of Intestinal Tumours*, 2nd edn. World Health Organization International Histological Classification of Tumours. Springer-Verlag: Berlin.

JEN J, POWELL SM, PAPADOPOULOS N, SMITH KJ, HAMILTON SR, VOGELSTEIN B AND KINZLER KW. (1994). Molecular determinants of dysplasia in colorectal lesions. *Cancer Res.*, **54,** 5523–5526.

JUNG S AND SCHLUESENER HJ. (1991). Human T lymphocytes recognize a peptide of single point-mutated, oncogenic *ras* proteins. *J. Exp. Med.*, **173,** 273–276.

KAHN SM, JIANG W, CULBERTSON TA, WEINSTEIN IB, WILLIAMS GM, TOMITA N AND RONAI Z. (1991). Rapid and sensitive nonradio-active detection of mutant K-*ras* genes via 'enriched' PCR amplification. *Oncogene*, **6,** 1079–1083.

KIM H, JEN J, VOGELSTEIN B AND HAMILTON SR. (1994). Clinical and pathological characteristics of sporadic colorectal carcinomas with DNA replication errors in microsatellite sequences. *Am. J. Pathol.*, **145,** 148–156.

LANDIS JR AND KOCH GG. (1977). The measurement of observer agreement for categorical data. *Biometrics*, **33,** 159–174.

MCDOUGALL CJ, NGOI SS, GOLDMAN IS, GODWIN T, FELIX J, DECOSSE JJ AND RIGAS B. (1990). Reduced expression of HLA class I and II antigens in colon cancer. *Cancer Res.*, **50,** 8023–8027.

MCLELLAN EA, OWEN RA, STEPNIEWSKA KA, SHEFFIELD JP AND LEMOINE NR. (1993). High frequency of K-*ras* mutations in sporadic colorectal adenomas. *Gut*, **34,** 392–396.

MAYER L AND SHLIEN R. (1987). Evidence for function of Ia molecules on gut epithelial cells in man. *J. Exp. Med.*, **166,** 1471–1483.

MIYAKI M, SEKI M, OKAMOTO M, YAMANAKA A, MAEDA Y, TANAKA K, KIKUCHI R, IWAMA T, IKEUCHI T, TONOMURA A, NAKAMURA Y, WHITE R, MIKI Y, UTSUNOMIYA J AND KOIKE M. (1990). Genetic changes and histopathological types in colorectal tumors from patients with familial polyposis coli. *Cancer Res.*, **50,** 7166–7173.

MÖLLER P, KORETZ K, SCHLAG P AND MOMBURG F. (1991). Frequency of abnormal expression of HLA-A, B, C and HLA-DR molecules, invariant chain, and LFA-3 (CD58) in colorectal carcinoma and its impact on tumor recurrence. *Int. J. Cancer*, Suppl. **6,** 155–162.

MOMBURG F, DEGENER T, BACCHUS E, MOLDENHAUER G, HÄMMERLING G AND MÖLLER P. (1986). Loss of HLA-A, B, C and *de novo* expression of HLA-D in colorectal cancer. *Int. J. Cancer*, **37,** 179–184.

MUTO T, BUSSEY HJR AND MORSON B. (1975). The evolution of cancer of the colon and rectum. *Cancer*, **36,** 2251–2270.

NORAZMI M, HOHMANN AW, SKINNER JM AND BRADLEY J. (1989). Expression of MHC class I and class II antigens in colonic carcinomas. *Pathology*, **21,** 248–253.

NORHEIM ANDERSEN S, ROGNUM TO, LUND E, MELING GI AND HAUGE S. (1993). Strong HLA-DR expression in large bowel carcinomas is associated with good prognosis. *Br. J. Cancer*, **68,** 80–85.

NOWELL PC. (1976). The clonal evolution of tumor cell populations. *Science*, **194,** 23–28.

PRETLOW TP, BRASITUS TA, FULTON NC, CHEYER C AND KAPLAN EL. (1993). K-*ras* mutations in putative preneoplastic lesions in human colon. *J. Natl Cancer Inst.*, **85,** 2004–2007.

ROGNUM TO, FAUSA O AND BRANDTZAEG P. (1982). Immunohistochemical evaluation of carcinoembryonic antigen, secretory component, and epithelial IgA in tubular and villous large bowel adenomas with different grades of dysplasia. *Scand. J. Gastroenterol.*, **17,** 341–348.

ROGNUM TO, BRANDTZAEG P AND THORUD E. (1983). Is heterogenous expression of HLA-DR antigens and CEA along with DNA-profile variations evidence of phenotypic instability and clonal proliferation in human large bowel carcinomas? *Br. J. Cancer*, **48,** 543–551.

ROGNUM TO, BRANDTZAEG P, ELGJO K AND FAUSA O. (1987). Heterogenous epithelial expression of class II (HLA-DR) determinants and secretory component related to dysplasia in ulcerative colitis. *Br. J. Cancer*, **56,** 419–424.

RUIZ-CABELLO F, LÓPEZ NEVOT MA AND GARRIDO F. (1988). MHC class I and II gene expression on human tumors. *Adv. Exp. Med. Biol.*, **233,** 119–128.

SANGER F, NICLEN S AND COULSEN AR. (1977). DNA sequencing with chain terminating inhibitors. *Proc. Natl Acad. Sci. USA*, **74,** 5463–5467.

SASAKI M, SUGIO K AND SASAZUKI T. (1990). K-*ras* activation in colorectal tumors from patients with familial polyposis coli. *Cancer*, **65,** 2576–2579.

SCOTT N, BELL SM, SAGAR P, BLAIR GE, DIXON MF AND QUIRKE P. (1993). p53 expression and K-*ras* mutation in colorectal adenomas. *Gut*, **34,** 621–624.

SOH K, YANAGISAWA A, HIRATSUKA H, SUGANO H AND KATO Y. (1993). Variation in K-*ras* codon 12 point mutation rate with histological atypia within individual colorectal tumours. *Jpn. J. Cancer Res.*, **84,** 388–393.

TSIOULIAS G, GODWIN TA, GOLDSTEIN MF, MCDOUGALL CJ, SING-SHANG N, DECOSSE JJ AND RIGAS B. (1992). Loss of colonic HLA antigens in Familial adenomatous polyposis. *Cancer Res.*, **52,** 3449–3452.

VOGELSTEIN B, FEARON ER, HAMILTON SR, KERN SE, PREISINGER AC, LEPPERT M, NAKAMURA Y, WHITE R, SMITS AMM AND BOS JL. (1988). Genetic alterations during colorectal-tumor development. *N. Engl. J. Med.*, **319,** 525–532.

YAMASHITA N, MINAMOTO T, OCHIAI A, ONDA M AND ESUMI H. (1995). Frequent and characteristic K-*ras* activation and absence of p53 protein accumulation in aberrant crypt foci of the colon. *Gastroenterology*, **108,** 434–440.